# Polymorphic variants in *DOCK7*, *ABCG8*, *UBE2E2*, and *SYN2* genes associated with type 2 diabetes in the Uzbek population

**DOI:** 10.3389/fcdhc.2025.1494128

**Published:** 2025-02-28

**Authors:** Darya Zakirova, Alisher Abdullaev, Dilbar Dalimova, Elina Aguryanova, Fazliddin Khonboev, Nilyufar Khushvakova, Nodira Alikhanova, Feruza Takhirova

**Affiliations:** ^1^ Biotechnology Department, Center for Advanced Technologies, Tashkent, Uzbekistan; ^2^ Department of Endocrinology, Samarkand State Medical University, Samarkand, Uzbekistan; ^3^ Diabetology Department of the Republican Specialized Scientific-Practical Medical Center of Endocrinology Named after Academician Y.Kh.Turakulov, Tashkent, Uzbekistan

**Keywords:** type 2 diabetes, Uzbek population, rs4299376, rs6780569, rs7616006, rs12749263, rs7957197, rs4402960

## Abstract

**Background:**

Diabetes is a leading cause of death, affecting nearly half a billion adults worldwide. With projections indicating a significant increase in prevalence, understanding the genetic factors that contribute to diabetes, particularly type 2, is crucial.

**Methods:**

This study investigated the association of specific polymorphisms with type 2 diabetes (T2D) in the Uzbek population. A total of 165 individuals, including 125 patients with T2D and 40 controls, were genotyped for variants located in the *DOCK7*, *ABCG8*, *UBE2E2*, *SYN2*, *HNF1A*, and *IGF2BP2* genes using real-time polymerase chain reaction.

**Results:**

The analysis revealed significant associations between these polymorphisms and T2D under various genetic models. The distribution of the genotype frequencies was consistent with the Hardy–Weinberg equilibrium.

**Conclusion:**

The findings of this study underscore the importance of ethnic and geographical diversity in genetic studies and contribute to the understanding of T2D in the Uzbek population. Further research is needed to explore the clinical implications of these genetic associations.

## Introduction

Diabetes is in the top 10 causes of death in adults (1). Nearly half a billion people live with diabetes, and projections indicate that this number will increase by 25% by the year 2030 and by 51% by the year 2045. It is estimated that 578 million individuals (10.2%) will have diabetes by 2030, and this figure is expected to increase to an astonishing 700 million (10.9%) by the year 2045 (2).

The genetic inheritance of diabetes has been well studied on a global scale; however, the ethnic characteristics of populations play a significant role in the inheritance of multifactorial diseases, including type 2 diabetes (T2D) (3–8).

In the quest to unraveling the genetic factors of diabetes, researchers have examined several polymorphic markers linked to cholesterol. Notably, polymorphisms such as rs4299376 and rs12749263 have undergone thorough investigations. The single nucleotide polymorphism (SNP) rs4299376, a variant of the *ABCG8* gene on chromosome 2, is associated with traits related to blood lipids and dyslipidemia, including the modulation of cholesterol levels and cardiovascular disease risk (9). Similarly, SNP rs6780569, which is situated within the *UBE2E2* gene on chromosome 3, has been implicated in T2D in diverse populations (10), including those of Japanese, Korean, and Chinese descent (11).

Furthermore, SNP rs7616006, located in the *SYN2* gene on chromosome 3, is linked to glycated hemoglobin levels, (12). On the other hand, SNP rs12749263, found in the *DOCK7* gene on chromosome 1, is associated with total cholesterol levels. In-depth studies on pancreatic islet function revealed that the *HNF1A* rs7957197 variant influences glycemic traits (13). Among the *IGF2BP2* gene variants, rs4402960 stands out due to its strong association with T2D, particularly in European and East and South Asian populations (14, 15).

As the distribution of allele and mutation frequencies in genes varies based on the geographical and ethnic characteristics (16), this study aimed to investigate the association of these polymorphisms with T2D, as well as its clinical presentation, in the Uzbek population.

## Methods

The search for potential variants associated with metabolic disorders, as well as related studies, was carried out in the Type 2 Diabetes Knowledge Portal (T2DKP), an open-access resource for human genetic information on T2D. Potential markers associated with insulin levels, lipid profiles, and metabolic disorders, as well as related studies, were searched in T2DKP (17). The identified markers are systematically evaluated in stages, with the current study focusing on a subset relevant to T2D in the Uzbek population.

This study included 165 people, of whom 125 were patients with T2D, aged over 45 years, of Uzbek nationality and 40 were controls. The patients were receiving outpatient care in the RSSP Medical Center of Endocrinology, named after the academician Y.Kh. Turakulov. During recruitment, each participant completed questionnaires covering a wide range of medical information and provided blood samples for DNA analysis. T2D was identified based on a fasting glucose level of ≥126 mg/dl and a glycosylated hemoglobin (A1c) value of at least 6.5%.

The exclusion criteria for the diabetic patients in the study were: type 1 diabetes mellitus, age under 45 years, and not of Uzbek nationality. The inclusion criteria for the diabetic patients in the study were: T2D mellitus, age over 45 years, and of Uzbek nationality.

### Statistical analysis

The phenotype and genotype data were entered into Microsoft Excel 2019 (Microsoft, Redmond, WA, USA) for initial processing. The interquartile range (lower quartile, 25%; upper quartile, 75%) and the median of the sample were calculated. A logistic regression analysis was conducted using the R programming language (version 4.2.2; R Core Team, Indianapolis, IN, USA) and the SNPassoc software package (The R Foundation for Statistical Computing, Vienna, Austria) to analyze statistically significant associations between the predicted genotype and the development of the disease via common genetic models (18).

### Genotyping

Polymorphic variants of the genes were identified with real-time polymerase chain reaction (PCR) using allele-specific primers, gene-specific primers, and DNA probes. Details of the analyzed polymorphic variants are listed in **Table 1**. PCR was performed using a QuantStudio 5 Real-Time PCR System (Applied Biosystems, Foster City, CA, USA). The reaction was carried using the TaqMan^®^ Genotyping Assay kit (Thermo Fisher Scientific, Waltham, MA, USA) following the manufacturer’s standard protocol.

**Table 1:** Characteristics of studied polymorphic variants and their association with type 2 diabetes in the Uzbek population.

Gene	Polymorphic variant	Location	Molecular consequence	MAF (GWAS)
*ABCG8*	rs4299376 [G>T]	2:43845437	Intronic	G = 0.3
*UBE2E2*	rs6780569 [G>A]	3:23156993	Upstream	A = 0.12
*SYN2*	rs7616006 [A>G]	3:12226148	Intergenic	G = 0.43
*DOCK7*	rs12749263 [T>C]	1:62562527	Intronic, upstream *ANGPL3*	C = 0.24
*HNF1A*	rs7957197 [T>A]	12:121022883	Intronic	A = 0.19
*IGF2BP2*	rs4402960 [G>T]	3:185793899	Intronic	T = 0.32

*MAF*, minor allele frequency; *GWAS*, genome-wide association study.

This research was carried out within the framework of the project FZ-202103029 supported by the Ministry of Innovative Development of Uzbekistan. The study was approved by the Ethics Committee at the RSRPMCC, and all study participants signed an informed consent. The study was conducted in accordance with the World Medical Association Declaration of Helsinki (19).

## Results

The ages of the patients with T2D included in the study ranged from 44 to 75 years (median = 60.5 years). The ages in the control group ranged from 46 to 74 years, with a median of 57 years. According to the Mann–Whitney *U* test, there were no statistically significant differences in the ages of the T2D patient group and the control group (*р* = 0.0536, *U* = 1,993). The difference in the body mass index (BMI) was statistically significant between the T2D patient group and the control group (**Table 2**). As shown in **Table 2**, there were statistically significant differences in the glycated hemoglobin levels between the two groups. The triglyceride (TG) levels of the cases were also significantly different from those of the controls (**Table 2**). In the Uzbek population, the high-density lipoprotein (HDL) levels were significantly higher in the control group compared with those in the patient group, while the low-density lipoprotein (LDL) levels did not demonstrate significant differences (**Table 2**).

**Table 2:** Investigation of the clinical data and medical history according to the Mann–Whitney *U* test.

Clinical data	Cases (T2D patients) Median (QL–QU)	Controls (healthy) Median (QL–QU)	Mann–Whitney *U*	
			*p*-value	*U*
Age (years)	60.5 (54–65)	57 (49–63)	0.0536	1,993
BMI (kg/m^2^)	29.7 (26.3–34.4)	26.3 (24.3–28.4)	<0.00001	1,469.5
A1c (%)	10.5 (8.9–12.3)	5.30 (4.7–5.8)	<0.00001	4,353.5
TG (mmol/l)	2.1 (1.5–3.4)	1.71 (1.10–2.87)	0.00652	3,464
HDL-C (mmol/l)	1.1 (0.9–1.3)	1.17 (1.1–1.3)	0.01828	1,739
LDL-C (mmol/l)	3.1 (2.6–3.8)	2.9 (2.5–3.7)	0.42952	2,161

*T2D*, type 2 diabetes; *BMI*, body mass index; *A1c*, glycated hemoglobin; *HDL-C*, high-density lipoprotein-cholesterol; *LDL-C*, low-density lipoprotein-cholesterol; *TG*, triglyceride; *T2D*, type 2 diabetes; *QL*, quartile lower (25th percentile); *QU*, quartile upper (75th percentile).

The results of the genetic analysis of the polymorphic gene variants in the groups of cases and controls in the Uzbek population demonstrated an association between the rs4299376 variant and T2D under the additive genetic model (*p* = 0.008375). An association between the polymorphic gene variant rs6780569 and T2D was also demonstrated under the additive genetic model (*p* = 0.021334). Moreover, an association was demonstrated between the polymorphic gene variant rs7616006 and T2D under the additive genetic model (*p* = 0.02316), as well as between the polymorphic variant rs12749263 and T2D under the same model (*p* = 0.006540). The genotype frequency distribution in all groups corresponded to the Hardy–Weinberg equilibrium (*р* > 0.05).

To eliminate false-positive results and to apply stricter significance criteria for the identified associations, Bonferroni correction was performed. After the correction, only two SNPs, i.e., rs4299376 and rs12749263, continued to demonstrate significant associations. Stratification by BMI revealed that, in the group of individuals with higher BMI (>25 kg/m^2^), a significant protective effect was observed, suggesting that the effects of rs4299376 (OR = 0.50, 95%CI = 0.29–0.88, *p* = 0.01732) and rs12749263 (OR = 0.47, 95%CI = 0.27–0.82, *p* = 0.007767) are more pronounced in this group. Stratification by age revealed that the effect of rs4299376 varied with age: in the 44- to 59-year age group, a stronger protective effect was observed (OR = 0.21, 95%CI = 0.09–0.47, *p* = 2.161e−05), whereas in individuals aged 60+ years, the association became positive, but not significant (OR = 1.47, 95%CI = 0.59–3.64, *p* = 0.3830). In contrast, for rs12749263, the effect remained protective across both age groups, with OR = 0.66 (95%CI = 0.35–1.25, *p* = 0.2030) for ages 44–59 years and OR = 0.36 (95%CI = 0.16–0.80, *p* = 0.01195) for ages 60+ years.

The polymorphic gene variants rs7957197 and rs4402960 did not demonstrate an association between the predictive genotype and the disease (*p* > 0.05) (**Table 3**).

**Table 3:** Association analysis of the polymorphic variants with type 2 diabetes in the Uzbek population.

Polymorphic variant	Risk allele (allele frequency in the Uzbek population)	Non-risk allele	Log-additive model		
			OR	95%CI	*p*-value
rs4299376	T = 0.20	G	0.52	0.32–0.84	0.008375
rs6780569	A = 0.12	G	3.25	0.99–10.64	0.021334
rs7616006	G = 0.41	A	1.80	1.06–3.05	0.02316
rs12749263	T = 0.19	C	0.50	0.31–0.82	0.006540
rs7957197	A = 0.09	T	1.01	0.45–2.26	0.9900
rs4402960	T = 0.34	G	0.83	0.51–1.34	0.4423

*CI*, confidence interval; *OR*, odds ratio.

## Discussion

To the best of our knowledge, little has been previously reported about the association of SNP rs4299376 with T2D mellitus. According to previous meta-analysis studies, the rs4299376 variant is associated with elevated levels of cholestanol, campesterol, and sitosterol and is a high absorption marker for cholesterol ratios (20). In addition, rs4299376 has been associated with all ratios of the absorption markers to lathosterol. *ABCG5* was significantly related to the level of total cholesterol (TC)-standardized serum sitosterol, a marker for intestinal cholesterol absorption. SNP rs4299376 showed a significant association with intestinal cholesterol absorption markers (20, 21). Diabetic dyslipidemia, which is often associated with T2D, indicates a harmful shift in lipid levels, marked by a reduced HDL (“good”) cholesterol and increased LDL (“bad”) cholesterol and TGs (22). It is also known that people with T2D often have higher levels of LDL (23). According to the results of the genetic analysis, the rs4299376 variant is associated with T2D under several genetic models (*p* < 0.05). It was hypothesized that the rs4299376 polymorphism could impact the development of T2D mellitus through its involvement in fat metabolism.

Another marker being studied is the rs6780569 *UBE2E2* gene, which encodes the ubiquitin-conjugating enzyme E2E2 that plays an important role in the synthesis and secretion of insulin (24). This also demonstrated an association with T2D in the Uzbek population under several genetic models. Ubiquitin is a small protein that forms covalent bonds with other cellular proteins, which leads to their degradation by proteasomes (25, 26). The ubiquitination cascade necessitates the orchestrated action of three distinct classes of enzymes: the ubiquitin-activating enzymes (E1), the ubiquitin-conjugating enzymes (E2), and the ubiquitin ligases (E3) (27). Contemporary research indicates that the suppression of proteasomal activity can indirectly attenuate the biosynthesis of insulin (25, 28) and thus influence the onset of diabetes. Other researchers have suggested that changes in the ubiquitin–proteasome system or autophagy could lead to the effect of insulin resistance and the development of T2DM (27). This has been confirmed by other studies. In particular, Kim et al. presented findings from a study that examined the association between variants of the *UBE2E2* gene (polymorphism rs6780569) and the risk of gestational diabetes mellitus among Korean women (29).

Two other polymorphisms (i.e., rs7616006 and rs12749263) were genotyped by us in the Uzbek population, wherein, in the context of a comprehensive genome-wide association study (GWAS), the polymorphism rs7616006 within the *SYN2* gene has been observed to exhibit a significant association with the incidence of diabetes mellitus, as well as with the quantitative levels of glycated hemoglobin (30). The polymorphism rs12749263 in the *DOCK7* gene has been associated with an elevation in BMI (31). Both of these polymorphisms also showed significant associations with T2D in the Uzbek population according to several inheritance models. These associations suggest that these genetic variations might play a crucial role in the pathophysiological mechanisms that underlie the onset and progression of T2D, thereby providing potential targets for therapeutic intervention and risk stratification in the affected population.

Two other polymorphisms, i.e., rs7957197 and rs4402960, did not demonstrate significant associations in the analysis. This could be attributed to the limited sample size or the population-specific genetic variability.

One notable limitation of this study is its relatively small sample size, which might have impacted the statistical power and generalizability of the findings. To address this, future studies should include a significantly larger and more diverse cohort in order to strengthen statistical power and provide more robust conclusions, as well as aim to stratify the population by age and sex. Particular attention should be given to the study of how genetic factors influence the early onset and progression of diabetes. In addition, this study did not use the most strongly associated markers reported through 17 years of GWAS of T2D, and there should be additional studies in the Uzbek population to enable the development of more predictive tools for the evaluation of the population risk of T2D from larger studies.

Furthermore, future investigations should build upon the findings of this study by integrating insights from prior genetic and epidemiological research and should leverage these data to refine hypotheses and design more targeted studies, incorporating advanced methodologies such as GWAS to dissect the complex interactions between genetic variants, demographic factors, and environmental influences. This approach will enable the identification of additional genetic loci associated with T2D, offering deeper insights into the genetic architecture of the disease within the Uzbek population.

## Conclusion

We confirmed that four out of the six studied polymorphic variants demonstrated a significant association with T2D in the Uzbek population. These are rs4299376, rs6780569, rs7616006, and rs12749263. These polymorphic variants can later be used as informative genetic biomarkers for identifying the risks of developing T2D in order to improve the diagnosis and the quality of treatment of patients with T2D in the Uzbek population. However, after applying Bonferroni correction, only two SNPs, i.e., rs4299376 and rs12749263, maintained significant associations, reinforcing their potential role as key genetic markers in the studied population.

## Data Availability

Genotyping data of control groups and patients with type 2 diabetes in the Uzbek population can be accessed via this link: 10.6084/m9.figshare.28449956. The additional material available upon request to interested researchers.
